# Case of Interest

**Published:** 1882-08

**Authors:** 


### ARTICLE IX.

### *Case of Interest.*

Dr. Rich, of New York, describes the following interesting case: "The subject was a daughter of an eminent physician of this city. Her teeth had been continuously under my care from childhood. She grew up a fine, healthy, robust girl, with large, regular, fine teeth, which she kept in good order and was very proud of. When she was about twenty years of age she complained of an occasional severe pain in the right superior first molar. She consulted me about the trouble the tooth was giving her, and I made a careful and thorough examination of the offending member, but could not discover any cause for the pain she was suffering. The tooth was perfectly sound, not a speck of decay about it, and not at all sore to pressure and percussion. The application of a current of electricity gave no indication of a dead pulp. The intervals between the periods of suffering became shorter, and within six months after its appearance it became continuous, and the torture from it was almost unbearable. She could not sleep except when under the influence of narcotics; she became extremely nervous and irritable, and the derangement of the system was general. She lost her appetite, and ran down in health and flesh very rapidly, and her condition became a source of great anxiety to her family. During all this time she was under the care of her father, a most skillful physician, who tried every means available to relieve her and enable me to save the tooth. The symptoms were something like those that occur when there are osseous formations going on in the pulp, and with the idea that possibly that may be the cause of suffering, I destroyed and extirpated the pulp; no such formations were found in it, and no relief followed the removal of the pulp. I then extracted the tooth. The pain and nervous irritation immediately disappeared and the patient rapidly recovered her health and good looks. When I looked at the tooth after taking it out, I found a part of the ruptured membrane was still attached to the palatal root; upon examining this under the microscope, I discovered three points of inflammation in different parts of the membrane that remained on the root, about the twentieth of an inch in diamettr. With this exception, the membrane had the appearance of being strong and healthy, and except in those spots, was entirely free from inflammation. From some previous experiences, these spots of inflammation, the general character of the case, suggested spicules in the socket. The day after the tooth was extracted, I made a very careful examination of the socket, and found not only the three that corresponded with the points of inflammation but discovered with the microscope a number of other small spicules in that and the sockets of the other roots.

A few months after the extraction of the tooth I have just mentioned, the same symptoms were developed in the first superior molar of the left side. In the case of this tooth, as soon as the symptoms were well ascertained to be identical with those attending the commencement of her suffering, the tooth was extracted, as the patient would not undergo repetition of the torture experienced with the first one. And by this process this young lady has lost four of her upper molars during the last five years, the first and second molars on each side. As yet, none of the other teeth have been affected in that way."—*Odontological Society of New York.*

